# Defining the ecological and evolutionary drivers of *Plasmodium knowlesi* transmission within a multi-scale framework

**DOI:** 10.1186/s12936-019-2693-2

**Published:** 2019-03-08

**Authors:** Gael Davidson, Tock H. Chua, Angus Cook, Peter Speldewinde, Philip Weinstein

**Affiliations:** 1 School of Agriculture and Environment, University of Western Australia, Stirling Terrace, Albany, WA 6330 Australia; 2 School of Population and Global Health, University of Western Australia, Perth, Australia; 3 Faculty of Medicine and Health Sciences, Universiti Malaysia Sabah, Kota Kinabalu, Sabah Malaysia; 4 School of Biological Sciences, University of Adelaide, Adelaide, Australia

**Keywords:** Malaria, Zoonotic, *Plasmodium knowlesi*, Anthropogenic land use change

## Abstract

*Plasmodium knowlesi* is a zoonotic malaria parasite normally residing in long-tailed and pig-tailed macaques (*Macaca fascicularis* and *Macaca nemestrina,* respectively) found throughout Southeast Asia. Recently, knowlesi malaria has become the predominant malaria affecting humans in Malaysian Borneo, being responsible for approximately 70% of reported cases. Largely as a result of anthropogenic land use changes in Borneo, vectors which transmit the parasite, along with macaque hosts, are both now frequently found in disturbed forest habitats, or at the forest fringes, thus having more frequent contact with humans. Having access to human hosts provides the parasite with the opportunity to further its adaption to the human immune system. The ecological drivers of the transmission and spread of *P. knowlesi* are operating over many different spatial (and, therefore, temporal) scales, from the molecular to the continental. Strategies to prevent and manage zoonoses, such as *P. knowlesi* malaria require interdisciplinary research exploring the impact of land use change and biodiversity loss on the evolving relationship between parasite, reservoir hosts, vectors, and humans over multiple spatial scales.

## Background

Numerous ecosystems across the globe have been altered anthropogenically by deforestation, pollution, agricultural expansion, road construction, dam building, mining, eutrophication, increased nitrogen fixation, urbanization and other activities, thereby creating conditions suitable for the emergence of infectious diseases [1, 2]. Changing patterns of land use may instigate contact between humans and both domestic and wild animals, and disturb population distribution and abundance [3]. As a result, many of the current emerging infectious diseases (EIDs) are zoonotic in origin and present serious public health concerns. The zoonotic parasite, *Plasmodium knowlesi*, is one such disease which threatens Malaysia’s malaria eradication policy [4]. Eradication policies for the human malarias focus on behavioural prevention and treatment practices based around insecticide-treated bed nets, indoor spraying, and drug therapy. The challenge with zoonotic *P. knowlesi* malaria is that the mosquito vectors do not enter dwellings to rest and feed. Traditional behavioural methods offer no easy options for breaking the transmission cycle. Furthermore, accurate diagnosis of the parasite requires molecular methods, and ways to reduce this time-consuming process are being investigated [5].

Infectious disease emergence has been described as a complex interplay between social and ecological systems over many scales, ranging from the molecular to the continental [6–8]. Ecological drivers operating at multiple spatial scales are altering transmission dynamics between pathogens, vectors and hosts [7]. Anthropogenic land use change reduces biodiversity, increases the contact between natural hosts, vectors, and humans, and alters the ecology in ways that may be favouring pathogen transmission to humans [9, 10]. A detailed examination of ecological changes at the local landscape scale in relation to the transmission and spread of *P. knowlesi* has been presented in the relevant paper [11]. However, this local landscape scale is nested within a complex array of interactions between social and biological systems ranging from the global scale, where pathogen phylogenetic history plays a role, down to the molecular level, where genetic factors influencing pathogen, vector, and host dynamics are crucial to emerging infection rates [7, 8, 12, 13]. For this reason, multi-scaled socio-ecological models of infectious disease may be effectively applied to *P. knowlesi* emergence in order to determining the complex drivers of its emergence [13].

### Drivers of the transmission and spread of *Plasmodium knowlesi* at different ecological scales

#### Evolutionary drivers of *Plasmodium knowlesi*

Non-human primates (NHPs) share malaria parasites with humans and host-switching is an ongoing evolutionary process. *Plasmodium knowlesi* contrasts with many other parasite species because it has the capability of infecting both immature and mature stage erythrocytes in humans, and infects both monkey and human hosts [14]. This unique capacity relates to differences in the multigene families and the proteins they encode for in the human and NHP *Plasmodium* species/strains. The genetic differences appear to be critical in determining current and potential hosts and host-switching capabilities [14]. The evolution of these differences has arisen over millennia, in parallel with the evolution of mammals. It is suggested that a single host-switch event originally brought the *Plasmodium* parasite across into mammalian hosts from sauropsid-infecting ancestors [15]. This leap occurred at least 64 million years ago (Ma) [16]. Alongside this host-switching came the specialization into *Anopheles* mosquitoes as the specific vector for mammalian hosts [17].

There are now six species of human malaria parasites; *Plasmodium falciparum*, *Plasmodium vivax*, *Plasmodium ovale* (*Plasmodium ovale wallikeri* and *P. o. curtisi*), *Plasmodium malariae* and *Plasmodium knowlesi* [14, 18]. At least 30 *Plasmodium* species cause infection in the NHPs [19, 20] infecting 53 host species over 25 genera [21]. Most *Plasmodium* parasites are constrained to phylogenetically related hosts that belong to the same family. The exceptions to this rule are *Plasmodium brasilianum* and *P. knowlesi*, which infect hosts from three and two families, respectively [21]. The natural hosts for *P. knowlesi* are macaques (*Macaca fascicularis* and *Macaca nemestrina*) as well as leaf monkeys (*Presbytis melalophos*) [21, 22] and now humans. Thirteen genes found in *P. falciparum* and *P. vivax* are not found in *P. knowlesi* and may be limiting the parasite’s transmission success in human hosts for now [23].

In Africa, the Laverania ‘subgenus’ [15] are seven tightly host-specific parasites found only in the great apes (such as chimpanzees and bonobos) but were still able to give rise to the recent (between 40 and 60,000 years ago) host-switch event of *P. falciparum* into humans [24–30]. In Southeast Asia, five NHP species predominate—*P. knowlesi*, *Plasmodium inui*, *Plasmodium cynomolgi*, *Plasmodium fieldi*, and *Plasmodium coatneyi* [31]. Globally, spillover from NHP malaria parasites into humans (albeit in low numbers), has been reported from Costa Rica [32] and Venezuela [33] with *P. brasilianum/P. malariae*, Brazil with *Plasmodium simium* [34, 35], Northeast Brazil with *P. brasilianum/P. malariae* [36], Malaysia with *P. cynomolgi* [37], and the Central African Republic with the *P. vivax*-*like* strain from the great apes [38].

The *P. brasilianum* genome is 99.7% identical to human *P. malariae* [39] and similarly, *P. simium* is considered genetically indistinguishable from *P. vivax* [40]. *Plasmodium brasilianum/P. malariae* is being considered as an anthropozoonoses, equally able to infect monkeys and humans given the opportunity [41]. It is hypothesized that *P. brasilianum* and *P. simium* are most likely the result of host-switching from *P. malariae* and *P. vivax* respectively, brought to the New World by European colonizers [42–44].

For *P. knowlesi*, it is still the subject of speculation whether it emerged along with *P. vivax* and other closely related species from an African origin as opposed to an archaic Asian origin [31, 42, 45, 46]. What is understood is that *P. vivax* and *P. knowlesi* (which both possess Duffy Binding Proteins for erythrocyte invasion) diverged somewhere between 18 and 34 Ma which approximates the split between their respective hosts, the apes (Hominoidea) and Old World Monkeys (Cercopithecidae) [16]. Phylogenetic analysis of CSP and MSP-9 gene sequences reveal that *P. coatneyi* and *P. knowlesi* have a very close relationship, forming one monophyletic group, all descending from one ancestor, even though they differ in many important ways biologically [47, 48]. This clade relationship between *P. coatneyi* and *P. knowlesi* has been corroborated using SSU rRNA genes by Chua and colleagues [49]. Another monophyletic group is comprised of *P. cynomolgi, Plasmodium simiovale, P. simium* and *P. vivax* and together both these groups belong to a well-defined and distant clade [47]. It has been suggested that *P. vivax* diverged from *P. cynomolgi* when it host-switched to humans [50, 51].

The Old World macaques brought *P. knowlesi* across into Southeast Asia when land bridges became available during glacial periods over the past several million years [52, 53]. A divergence occurred between *P. knowlesi* and its closest ancestor, *P. coatneyi*, around 98,000–478,000 years ago in this region [54] indicating that the parasite predates human settlement in SE Asia [55]. *Plasmodium knowlesi* parasite numbers increased significantly 30,000–40,000 years ago [54], but whether this is connected to an increase in primate hosts or mosquito vectors is still undetermined [56]. According to data on population growth and stabilization of the long-tailed macaques on Borneo, their population increase occurred prior to the population expansion of *P. knowlesi* on the island [57].

Given the geographical separation of the archipelagic part of Southeast Asia, it is hypothesized that different locations will host different genetic variants of the *P. knowlesi* parasite [58, 59]. Most human infections of *P. knowlesi* are dominated by single genotypes from one of three main genomic clusters that show evidence of recent strong selection [60, 61], two clusters from each of the macaque hosts in Borneo (*Macaca fascicularis* and *Macaca nemestrina* Borneo) and a third cluster from Peninsular Malaysia found in humans [60, 62–65]. Geographical separation of the island of Borneo from the peninsular by sea-level rise at the end of the last ice age would have prevented macaque movement and been responsible for any allopatric divergence [66].

However, recent genomic analysis has uncovered chromosomal-segment exchanges between these subpopulations [61]. Benavente and colleagues [61] investigated the *P. knowlesi* genotype from Cluster 1 (Mf-Pk Borneo) and found two geographic subgroups corresponding to different geographical regions: the Kapit and Betong regions in Sarawak. Chromosome 8 of the genome presented an anomaly where distinct genetic differences were noted for this chromosome between the two geographical locations. Chromosome 8 contains information relating to interaction with the vector during the parasite life cycle. The researchers found that many of the *Macaca fascicularis/P. knowlesi* Betong genotypes had segments on chromosome 8 almost identically matching the chromosome 8 segments from the *Macaca nemestrina/P. knowlesi* Cluster 2 genotype [61]. This finding suggests a recent genetic exchange between these two macaque clusters which appear to be under strong selective pressure, given that 73% of the Betong subgroup share this new haplotype and 33/60 genetic isolates examined showed evidence of genetic exchange between these two clusters [61]. The parasite is, therefore, adapting to the two macaque hosts with enough genetic differentiation to be considered as subgroups, but still capable of recombining when contact arises. Benavente et al. [61] suggest that the parasites genetic diversity and the changing environment in Borneo may assist *P. knowlesi* with further host transitions in the future.

### Bioregional/regional drivers—distribution of vectors, hosts, and the influence of land use

For now, transmission of *P. knowlesi* malaria is restricted to the Leucosphyrus Group of *Anopheles* mosquitoes because the sporozoites of the parasite are unable to invade the salivary glands of other mosquito species [67]. This vector group restricts *P. knowlesi* to the Southeast Asian biogeographic region [68]. The Leucosphyrus group consists of two different complexes made up of many different species, some of which have been identified as important for carrying *P. knowlesi*: the Dirus Complex of *Anopheles dirus* and *Anopheles cracens*, and the Leucosphyrus Complex of *Anopheles latens*, *Anopheles balabacensis* and *Anopheles introlatus* [59]. The Leucosphyrus Complex vectors are found in Malaysia, Indonesia, Singapore, Brunei and parts of the Philippines while the Dirus Complex vectors are widespread in the northern mainland countries of Myanmar, Thailand, Cambodia and Vietnam [59]. The Leucosphyrus group of mosquitos feed on both monkeys and humans, with species such as *An. cracens*, showing a clear preference for humans [69–72].

*Anopheles cracens,* although from the Dirus Complex, is the primary vector of *P. knowlesi* in Peninsular Malaysia. It shares ten olfactory genes with *Anopheles maculatus*, the species responsible for human malaria in this region, but additionally carries a further five olfactory genes thought to be responsible for its ‘monkey-seeking’ biting behaviour [72, 73]. This mosquito is more commonly found in forest and farms than in larger settlements [74], but will enter villages, although not into dwellings. It prefers biting humans over monkeys at a ratio of 2:1 with peak feeding between 7 and 9 p.m. [69–72]. *Anopheles latens*, the vector of *P. knowlesi* in Kapit, Sarawak, is predominately detected in farming plots and forest locations, with highest infection rates occurring in the forest in humans engaged in forest-based activities. However, its propensity to transmit the parasite is highest in farms [75].

*Anopheles balabacensis* is the principal mosquito species transmitting the *P. knowlesi* parasite in Sabah [76–78]. In Kudat district, Sabah, for example, this vector comprised 94.4% of the total mosquito collection with highest densities found in villages over forest or farms [77]. This species was five times more commonly collected from the peri-domestic environment than from inside the dwelling [79] indicating its exophilic nature. However, the highest infection rates still occur in the forest and farming plots, possibly because of the proximity to macaques [79].

Chua and colleagues [49] examined all captured *An. balabacensis* from the Kudat district, Sabah, for Plasmodium infection and found that although this species comprised 89.87% (n = 1586) of the total catch, only 23 *An. balabacensis* mosquitos carried simian Plasmodium infections. *Plasmodium knowlesi* was found in only two mosquitoes (in combination with *P. inui* in one and with *P. cynomolgi* in the other), almost 9% of all Plasmodium infections. The close genetic relationship between the *P. knowlesi* parasite found in the human cases from Kudat, previous macaque isolates and the *An. balabacensis* mosquito suggest that infection is largely contingent on the mosquito having access to a reservoir of hosts [49].

The natural hosts for *P. knowlesi* are the cynomolgus monkeys, the long-tailed and pig-tailed macaques (*Macaca fascicularis* and *Macaca nemestrina,* respectively) found throughout Southeast Asia. These two species coexist throughout much of this region, however, at a more local level they show preferences for wetter alluvial riverine terrain compared to a drier hilly terrain respectively [80]. As *Macaca fascicularis* is a tree-traveller and *Macaca nemestrina* travels along the ground, attributes of the different habitats may explain their segregation within their natural habitats. Macaca fascicularis has a preference for denser canopy foliage with fewer canopy gaps [80]. The pig-tailed macaque has an upper distribution boundary around the Surat Thani-Krabi depression in Thailand [81]. North of this boundary, this macaque is considered as a different species, *Macaca leonina* [81]. There is evidence that *Macaca leonina* may also be a putative host species for the parasite given that positive *knowlesi* infection has been confirmed from the Yunnan province of China and coincides with the northward range of *Macaca leonina* into Myanmar, well beyond the range of the two main host species [82, 83]. This species also shares a close genetic relationship to *Macaca nemestrina* [59].

*Plasmodium knowlesi* infections have been detected in macaques by molecular methods from both Borneo and Peninsular Malaysia as well as Singapore and Thailand [54, 70, 84]. The highest prevalence of malaria positive macaques has been reported in the Kapit Division of Sarawak, with 87% of 83 long-tailed macaques and 50% of 26 pig-tailed macaques found to be *P. knowlesi* positive [84]. In Selangor, Malaysia, 60% of the wild macaques were found to be infected with the parasite [85]. Monkeys infected with *P. knowlesi* are naturally asymptomatic with low level parasitaemias [22, 86].

Risk-assessment maps for the parasite have been produced based upon ranges of the host species, vectors, land use patterns and confirmed infections in humans, macaques and mosquitos [58, 59, 82]. *Plasmodium knowlesi* risk exists for an arc of habitats reaching from Taiwan in the north, down through the archipelago of the Philippine and Indonesian islands, up the Malay peninsula and into the mainland of Southeast Asia onto Myanmar [67]. Taiwan has no recorded cases, although the same vector species does reach this far north and various macaque species also inhabit the forest and forest fringes of the island [67].

*Plasmodium knowlesi* became noted as a parasite of concern after Singh and colleagues [87] published their findings in 2004 on a cluster of cases from Kapit, Sarawak between 2000 and 2002. They identified *P. knowlesi* as the malaria parasite responsible for infection in 58% (n = 208) of the cases, as opposed to *P. malariae,* as had been previously diagnosed. Similar findings have been reported in other countries of the region. In Thailand, blood samples from as far back as 1996 found *P. knowlesi* to be responsible for malaria (largely as a co-infection with the human malarias) in 0.48% (n = 210) of the human malaria cases [88] and more recently in 0.6% (n = 1874) of cases in 2006–2007, [89], and in 0.67% (n = 3770) of cases from 2008–2009 [88]. In the Philippines, two cases were detected in 2006 [90] and in southern Myanmar, *P. knowlesi* comprised 21.9% (n = 146) of cases in 2008, also mainly as a co-infection with the human malarias [91]. Further confirmed human cases of the disease have now been recorded from Singapore [92, 93], Vietnam [94, 95], Cambodia, southern Thailand [96], Indonesia [97–99], Indonesian Borneo [100–102], Brunei, Myanmar [84, 91, 96, 103], the Nicobar and Andaman Islands of India [104], and quite recently from Laos [105].

The reality is however, that the true prevalence of *P. knowlesi* in the Southeast Asian region is difficult to estimate, and when or where this parasite first caused a natural infection in a human is unknown. The first reported infection in a human was 1965 from peninsular Malaysia [106] and presumptively again in 1971 from the same region [107]. Confirmed human cases are increasing, especially in Malaysian Borneo, although this may simply relate to increased awareness of, and testing for, this pathogen. There is an urgent need for cross-sectional studies that can provide information on patterns of infection and exposure at the community level to assess the true threat of this parasite [82, 108].

With reference to shifts in vector and host habitats, the Southeast Asian region has undergone dramatic changes in land use patterns. The economic pressures behind such transitions relate in part to global commodity markets driving consumption of palm oil and wood which are central to Malaysia’s economic growth [109–111]. The demand for palm oil is increasing by an estimated 15% per year [112] with Malaysia’s production expected to rise to 23.4 million tonnes by 2020 up from current production of 15 million tonnes [110]. Oil palm is considered a threat to biodiversity in Southeast Asia because these plantations are almost totally devoid of forest-dwelling animal species [113]. Malaysian Borneo has been severely impacted by deforestation over the past several decades and estimates suggest that 80% of the country had been logged and cleared for agriculture by 2009 [114]. Since this time, deforestation has continued at a rate of 350,000 ha each year [110]. Deforestation and land-use change may be influencing the distribution of *P. knowlesi* host and vector populations.

These land use changes have contributed to a different relationship between disease risk and environmental variables for Malaysia in comparison to continental Asia [59, 82]. Moyes and others [59] found that in the southern countries of Malaysia, Indonesia, Singapore, Brunei and part of the Philippines, there is a risk to humans for *P. knowlesi* from vectors of the Leucosphyrus complex being found together with macaque hosts within disturbed forest areas, including plantations, vegetable gardens and logging concessions. When members of the Dirus complex overlap with the habitat of the long-tailed macaques (*Macaca fascicularis*) in the northern endemic countries, the possibility for co-occurrence exists for savannah, vegetation mosaics, and cropland as well as forested areas [59]. In Malaysia, deforestation and changes in land use have induced macaques to move away from forest to farms and semi-urban areas, with the Leucosphyrus mosquitos therefore also extending their range to become more predominant in farms, villages and areas of logged forest [74, 115].

Current knowledge suggests that the parasite may be adapting to these changes in the distribution and vectorial capacity of the mosquitos from the Leucosphyrus Complex. Benavente et al. [61] reported on the strong selective pressure on the parasite being imposed by the current vectors within the Malaysian region. For example, one recombinant gene in chromosome 12 of *P. knowlesi* (*PKH_120710*) is orthologous to the gene (*Pfs47*-like) in *P. falciparum* which is known for its role in infecting *Anopheles gambiae* without activating its immune system [116]. A haplotype alteration in *Pfs47* in *P. falciparum* renders it compatible with a different species of mosquito [116]. Benavente et al. [61] found that close to half of the *P. knowlesi* isolates taken from the Betong region of their study in Sarawak presented a recombinant profile in this gene indicating the potential for these isolates to adapt to other mosquito vectors. Thus, other more ecologically widespread mosquitoes may be only ‘one mutation event away’ from being able to act as vectors, potentially increasing the range of the parasite considerably [67].

#### Individual-level drivers

Individual level drivers of emergence relate to the human characteristics and behaviours that bring humans into contact with the natural hosts and vectors of the parasite [7]. Gaining an understanding of the breadth and clinical severity of infection from *P. knowlesi* is hampered by the need for molecular methods for accurate diagnosis. Polymerase chain reaction (PCR) methods are accurate but not feasible in most malaria endemic regions because of the cost of the equipment and the training required. Also, they do not provide the rapid results needed for treatment selection. However, a novel non-PCR method called loop-mediated isothermal amplification (LAMP) is showing promise for accurate and fast diagnostic results with low technology requirements [117]. Microscopy of stained blood smears and rapid diagnostic immunochromatographic tests (RDTs) are used widely and successfully for the detection of the other four human malarias [118], but are inadequate when attempting to diagnose *P. knowlesi* at the necessary levels of sensitivity and specificity [117, 119, 120]. For now, in the *P. knowlesi* endemic areas, any malaria infection which appears as *P. malariae* through microscopy is considered as *P. knowlesi* and treatment provided accordingly [121].

In Malaysian Borneo, those most at risk of contracting *P. knowlesi* infection have been noted to be males and either traditional subsistence farmers or those working in jobs relating to agriculture and forestry [108, 122, 123]. Data from 2016 for Sabah and Sarawak show that *P. knowlesi* infected more men than women (80%) with most cases occurring in adults, especially those aged over 55 years [78]. In contrast to these findings, a recent prospective study from Sabah reported a median age for *P. knowlesi* cases to be 33 years (IQR, 21–49 years) yet still predominately male [124]. The presence of macaques in proximity to human populations increases the risk of infection [49]. High levels of forest clearing together with remaining forest cover near houses is associated with *P. knowlesi* exposure, with the creation of such forest fringes known to encourage macaques [108]. Males may be more prone to clinical infection due to a higher number of infective bites resulting from proximity to macaques in these work-related areas [108].

The emerging possibility that *P. knowlesi* is being transmitted from human to human certainly exists, but to date no evidence has been found [125]. For human to human infection, the parasite would need to differentiate into the sexual gametocyte stage within the host red blood cell [126]. *Plasmodium knowlesi* gametocytes have been detected in human infections in Malaysia using microscopy [127] and reverse transcription (RT-PCR) methods to measure the level of *pks25*, an mRNA gene that is expressed in mature gametocytes [94, 128].

Although studies have generally shown a lack of case clustering in human settlements [129], Fornace et al. [130] and Barber et al. [131] both found family case clusters in districts in Sabah with a wide age distribution of infection (children < 15 years as well as women), including one infected family that had not travelled outside of Kudat township. In Aceh, Indonesia, a family cluster of cases (mother and two teenagers) occurred in a residential location nearby to the forest, indicating infection from the peri-domestic area [97]. Manin et al. [79] emphasize the importance of peri-domestic exposure from their research in Sabah where *An. balabacensis* was found to be 5.5-fold more abundant outside of dwellings in the early evenings than inside them. The lack of clustering of parasite genotypes in humans or macaques is further suggestive that transmission is zoonotic [54, 63, 64] However, according to Yakob et al. [132], this scenario could also be a result of human outbreaks that remained relatively small and localized, with transmission chains that did not extend far beyond the immediate population.

To estimate community prevalence of the knowlesi parasite in endemic areas, serological investigations were undertaken in three communities: two from northern Sabah and another from the Philippines. Results showed that women were similarly exposed to *P. knowlesi* as men, but with a higher proportion of subclinical outcomes. Fornace et al. [108] also found *P. knowlesi* exposure in children under 5 years of age at all three study sites, suggestive of low-level ongoing transmission. In Kudat province, Borneo, *P. knowlesi* is the most common cause of childhood malaria [133]. In general, individuals exposed to *P. knowlesi* were younger than those with sero-prevalence for the non-zoonotic malarias. Subclinical infections and exposure have therefore been found in populations in Sabah [108, 130], Northern Sumatra [98], Sarawak [134], and the Philippines [108] and these individuals may be contributing to the transmission of *P. knowlesi* malaria to others.

Co-infection with *P. knowlesi* and the human malarias is relatively common [70, 87, 89, 98, 108, 135] and may indicate human to human transmission where the vectors pick up *P. knowlesi* from humans [94]. In the border region between China and Myanmar, Jiang and colleagues [91] found that *P. knowlesi* was most commonly found as a co-infection with either *P. falciparum* (13%) or *P. vivax* (13%) rather than as a mono-infection (4%). In Khanh Phu, southern Vietnam, sampling over 2009–2010 found *P. knowlesi* infections in 26% of people sampled (32/125) and always as a co-infection predominantly with *P. vivax* followed to a much lesser degree by *P. falciparum* [136]. In a more recent study from Khan Phu, Vietnam, 70% of mosquitoes tested that had *P. knowlesi* in their salivary glands also carried the human malaria parasites, *P. falciparum* and/or *P. vivax* [94].

Agencies in Malaysia have been efficient in reducing the incidence of the two main human malarias in the region: *P. falciparum* and *P. vivax*. However this situation may be assisting *P. knowlesi* in the evolutionary transition into humans [137]. It is believed that *P. falciparum* and *P. vivax* may be outcompeting *P. knowlesi* within the human host as these parasites are so well adapted now to humans [137]. In areas such as Sabah and Sarawak, Borneo, where the prevalence of human malaria parasite is in decline, *P. knowlesi* may have a better chance of infecting human hosts, therefore, resulting in increasing incidence in this region.

Imai and colleagues [138] argue that there is a very low chance of sustained human to human transmission in the absence of macaques, with an estimated R_o_ = 1.04 in one of their models. However, continuing and closer contact between humans and macaques may alter mosquito preference in the future and lead to increased human to human transmission [67]. According to the Ross-McDonald mathematical model for malarial transmission, even a small change in preference for humans exerts a sizeable impact upon malaria transmission because of the exponential nature of this particular variable within the model [139]. Yakob et al. [140] used mathematical modelling to demonstrate that as humans increase their contact with the knowlesi parasite, more anthropophilic vectors will be selected for, as will a parasite that has a preference for human blood meals. These conditions may occur if the population density of humans in any given endemic area exceeds that of the macaques and a tipping point is reached.

### Drivers at the cellular and genetic level

Drivers of *P. knowlesi* transmission operating at these micro-scales relate to pathogen exposure, immunity, and health status of the human hosts. Together with these cellular factors, the different genotypes of *P. knowlesi* and invasion mechanisms have an important role to play in infection outcomes and continuing adaptation to humans as hosts [7]. Coevolution of malaria parasites and humans has resulted in a delicate balance between the levels of parasitaemia developed and the immune system of the host, with natural selection favouring maximum parasite transmission and limited host damage [141]. An important goal of malaria research is to better understand how the genomic differences in the *Plasmodium* parasites confer adaption for specific hosts and the different erythrocyte developmental stages [14].

Lim et al. [142] has established that primate-adapted *P. knowlesi* multiplies poorly in human blood because of its affinity for reticulocytes. In vertebrate blood, reticulocytes (1% of blood cells) are immature red blood cells still undergoing active protein synthesis of haemoglobin which takes place over several days until the cells mature and are classified as normocytes (99% of blood cells) [143]. In vitro testing of *P. knowlesi* in human blood found that over time the parasite quickly adapts to invade normocytes as well [142]. *Plasmodium knowlesi* contains proteins referred to as *NBPXa* and *NBPXb* (normocyte binding-like proteins-NBPs) which have been found essential for the invasion of human erythrocytes [144, 145]. The parasite’s short multiplication time (quotidian cycle) and its ability to infect both immature and mature erythrocytes results in hyper-parasitaemia developing quickly (within several days) if the disease goes undetected [146–148]. Respiratory distress along with jaundice, hypotension, acute renal failure, and organ dysfunction are typical of the severe complications manifested, with thrombocytopaenia nearly universal in cases of infection [146, 149, 150].

Disease virulence appears to relate to the *NBPXa* allele where patients in Sarawak infected with *NBPXa* had different disease markers (i.e. renal dysfunction) to those infected with *NBPXb* [151]. The allele is dimorphic and one of these forms (KH195) is more virulent than the other form (KH273) and responsible for the high levels of parasitaemia found in some cases [151, 152]. Investigations in Sabah and Peninsular Malaysia to compare all three geographical areas found that three distinct types of *P. knowlesi* exist: *NBPXa* (Type-1 and Type-2) throughout Malaysia with the existence of a third type (Type-3) detected only in Peninsular Malaysia [153]. All three types showed dimorphism (with the virulent and less virulent forms) indicating that virulent disease progression is also possible in Peninsular Malaysia [153], although far less common [154]. Whether these different types represent sub-species would require further genetic analysis [153]. Disease severity may also relate to a decreased capacity of the *P. knowlesi*-infected erythrocytes to deform (to pass through a capillary wall) thereby impeding microvascular flow and leading to severe symptoms, such as impaired organ perfusion, as occurs with *P. falciparum* [155].

*Plasmodium knowlesi* employs antigenic variation similarly to *P. falciparum*, to systematically alter antigens presented to the host’s immune system, thereby ‘hiding’ and leading to chronic infection [156–158]. A further conserved erythrocyte invasion function of *P. knowlesi,* equivalent to the tether structure of *P. falciparum,* involves protein trafficking where the knowlesi parasite generates membranous structures to modify the host erythrocyte for improved protein transport and parasite nutrition [159]. Three tryptophan-rich antigens (PkTRAg) also show binding to human erythrocytes and demonstrate a sharing of human erythrocyte receptors with *P. falciparum* in in vitro cultures [125].

A pathway that may act to currently restrict the *P. knowlesi* parasite in humans is the sialic acid pathway [51]. Sialic acid is a product released by mammals from hydrolysis of brain glycolipids or salivary mucins [160]. The most common forms are N-acetyl-neuraminic acid (Neu5Ac) and N-glycolyl-neuraminic acid (Neu5Gc) [160, 161]. Humans are unable to produce Neu5Gc from Neu5Ac due to a mutation in the required gene, CMAH, and therefore human erythrocytes express only Neu5Ac. In contrast, this gene remains active in the Old World monkeys, who have retained their ability to express Neu5Gc [160, 161]. *Plasmodium knowlesi* is unique in its ability to infect Neu5Gc positive macaques as well as Neu5Ac positive humans [84]. Two ligands of the Duffy binding proteins (similarly found in *P. vivax*), PkDBPβ and PkDBPγ, invade macaque erythrocytes using Neu5Gc, but a laboratory-developed line invades human erythrocytes using PkDBPα independently of Neu5Gc receptors, demonstrating a tolerance for the Neu5Ac variant in humans.

## Discussion and implications

Factors that influence the emergence of zoonotic *P. knowlesi* are parasite-host evolutionary dynamics, diversity, abundance and range of host and vector species, and the spatial and temporal overlap of contact between them [8]. These factors are in turn influenced by socio-ecological processes such as population growth, anthropogenic land use change, globalization of agricultural markets, and climate change [8]. These influences impact upon natural habitats and ecosystem dynamics at multiple scales in ways that alter microclimatic conditions, contact between parasite, host, and vector, and, sometimes, create new parasite-host dynamics with humans [6].

This connectivity with eco-social processes requires an interdisciplinary approach to enhance our understanding of the feedback loops involved with infectious disease emergence and spread [8]. In the case of *P. knowlesi*, there are many gaps in knowledge that need addressing, as outlined in Table 1. At the level of global phylogenetic analysis of malaria parasites, the ecological and evolutionary events that have led to host-switching and species diversification are not well understood, but are important to unravel for a greater knowledge of the disease in humans [15, 17]. The low occurrence of spill-over into humans from the NHP malaria parasites seems to show a tight host-specificity where significant ecological and molecular hurdles must be overcome to cross species [51]. For example, even though chimpanzees share > 99% genetic similarity with humans, the main malarial parasite infecting chimpanzees, *Plasmodium reichenowi*, does not infect humans and conversely *P. falciparum* only causes subclinical infection in them [162]. For *P. knowlesi*, more information is required on its phylogenetic history, life-history traits and host usage [15] to determine how it has evolved the capacity to spill-over into humans so readily now.

**Table 1:** Multi-scale ecological and evolutionary drivers of *Plasmodium knowlesi*. Adapted from Estrada-Pena et al. [7]

Scale and description	Drivers	Gaps in knowledge
Continental: global spread and dispersal of pathogen	Earth history	Phylogenetic links between *P. knowlesi* and other related taxa, shared life-history traits
Regional/biogeographic: broad climatic variation or geographical boundaries restrict *P. knowlesi* to SE Asia	Barriers to dispersal; natural distribution of hosts and vectors and biodiversity patterns	Distribution of the hosts and vectors of the parasite and the enabling and limiting factors for this distribution
Local landscape: fine-scale distribution of species within the habitat	Interaction between parasite, host, and vector within a rapidly changing habitat	Changing forest cover and land use and the effects on the distribution of vectors and hosts of *P. knowlesi* and parasite transmission rate
Individual: limiting and facilitating factors of transmission to humans	Individual health, behaviour, interaction with the vector and host, level of detection and treatment	Severity of infection in Malaysian Borneo versus the rest of SE Asia; asymptomatic carriage of the parasite within communities; degree of human to human transmission
Cellular: disease pathways	Infection route, immunological status, previous exposure	*P. knowlesi* multiplication in human blood; methods for accurate diagnosis
Molecular: human resistance to the disease; different genotypes of the parasite	Human host resistance or susceptibility; different *P. knowlesi* genotypes have varying disease severity and host preferences	Genetic factors influencing disease severity, genetic factors influencing choice of hosts and vectors, erythrocyte invasion pathways

More information is needed on the distribution and abundance of reservoir hosts and vectors of the parasite and how these relate to the rapid landscape-scale changes occurring in many parts of Southeast Asia, and notably in Borneo [163]. The first case of *P. knowlesi* infecting a human occurred during the 1960s in West Malaysia when most of the interior of the country was virgin forest and the mosquito vectors and humans had little contact [164]. As emphasized using this multiscale approach, the ecological situation in Malaysia today is very different. The vector species, along with the macaques can be found at the forest fringes, and have begun to encroach into farms and peri-domestic areas around villages [74, 79, 122]. Landscape scale ecological changes and potential links to *P. knowlesi* transmission in Malaysian Borneo have been described in the relevant paper [11]. There is a shortage of information on community exposure patterns as most data on *P. knowlesi* comes from reported cases of clinical infection [163]. In order to build models of transmission and spread for the parasite, wide geographical data is needed on community exposure levels, distribution of hosts and vectors, and associated ecological factors [108, 129, 163]. This information would assist with determining where hotspots of transmission are occurring and inform strategies for prevention and treatment [163].

Currently, human infections appear to be a result of human–macaque proximity [49, 165]. However, the potential exists for *P. knowlesi* to be transmitted from human to human, or to adapt to utilizing other NHPs as hosts, or other mosquito species as vectors [61, 138, 166]. As subclinical infection and exposure in humans is being more readily detected [108, 130], there is a need to better understand and monitor which subpopulation of the parasite is responsible and any genetic changes that are occurring [65]. The parasite has evolved into different strains adapted to the two different macaque hosts on Borneo and which have also diverged from the isolates in Peninsular Malaysia found in humans [60]. Historically, this emergence of three distinct genetic subpopulations in Malaysia occurred through genetic isolation, but currently recombination is occurring between the different macaque isolates. This process may be accelerated as their habitats are lost to deforestation and competition for resources forces them together [61]. This genetic recombination provides compelling evidence that rapid ecological change occurring on the island of Borneo may be furthering the parasites ability to host switch. Increased contact with humans also provides evolutionary opportunity for erythrocyte invasion pathways such as the sialic acid-independent pathway using of the Duffy-binding protein PkDBPα, or the tryptophan-rich antigens (utilized similarly by *P. vivax* and *P. falciparum*) to be exploited by *P. knowlesi* [125, 161].

This review had demonstrated how the evolution and biology of vectors, hosts, and pathogen, the changing landscape and ecology, and human behaviour, all interact at multiple ecological scales to produce the emerging disease risk that is now apparent for *P. knowlesi* [167]. The transmission and spread of *P. knowlesi* and its potential for host-switching needs to be considered with an interdisciplinary approach that incorporates the multi-scaled influences upon its emergence [7, 8, 10]. If any of the ‘host-switching’ events were to occur, there could be an increase in transmission and spread of this parasite, and a future in which *P. knowlesi* poses an even greater threat to population health.

## Abbreviations

NHPs: non-human primates
Ma: million years ago
PCR: polymerase chain reaction
NBPs: normocyte binding-like proteins
